# Na+/K+-ATPase: a multifunctional target in type 2 diabetes and pancreatic islets

**DOI:** 10.3389/fimmu.2025.1555310

**Published:** 2025-02-19

**Authors:** Lisha Mou, Zhenkun Fu, Tony Bowei Wang, Yuxian Chen, Ziqi Luo, Xinyu Wang, Zuhui Pu

**Affiliations:** ^1^ Department of Endocrinology, Institute of Translational Medicine, Shenzhen Second People’s Hospital, The First Affiliated Hospital of Shenzhen University, Guangxi University of Chinese Medicine, Shenzhen, Guangdong, China; ^2^ MetaLife Lab, Shenzhen Institute of Translational Medicine, Shenzhen, Guangdong, China; ^3^ Biology Department, Skidmore College, Saratoga Springs, NY, United States; ^4^ Imaging Department, The First Affiliated Hospital of Shenzhen University, Shenzhen Second People’s Hospital, Shenzhen, Guangdong, China

**Keywords:** diabetes, type 2 diabetes, sodium-potassium ATPase, islet, β-cells, therapeutic target, gene therapy, stem cell therapy

## Abstract

Type 2 diabetes (T2D) is a widespread metabolic disorder marked by hyperglycemia, arising from insulin resistance and relative insulin deficiency. This review investigates the critical role of Na+/K+-ATPase (NKA), a transmembrane protein essential for maintaining cellular ion gradients, in the pathophysiology of T2D. We provide an overview of NKA’s biological functions, emphasizing its involvement in cellular signaling pathways, insulin secretion, and glucose homeostasis. The potential of NKA as a therapeutic target for T2D is analyzed, showcasing innovative strategies such as NKA activators, gene therapy, and stem cell therapy aimed at enhancing NKA activity to achieve better glycemic control. Additionally, NKA’s multifunctional role in maintaining cell viability and modulating immune responses in islet transplantation may offer potential benefits for improving transplant outcomes. By elucidating the complex interactions between NKA and T2D, this review aims to shed light on developing novel therapeutic interventions that meet the multifaceted needs of individuals suffering from this chronic condition, ultimately improving their health outcomes.

## 1 Introduction

Type 2 diabetes (T2D) is the most prevalent form of diabetes (1), affecting millions of people worldwide and representing a significant global health challenge (2, 3). Despite efforts, T2D is complex to manage, and there’s a need for more effective treatments targeting its underlying causes (4). Understanding the pathophysiology of T2D is essential for developing effective therapeutic strategies (5). Among the various proteins involved in glucose metabolism and insulin signaling, Na+/K+-ATPase (NKA) plays a crucial role in maintaining ionic balance across cell membranes, which is vital for proper cellular function (6). NKA is responsible for the active transport of sodium out of cells and potassium into cells, processes that are critical for maintaining membrane potential and facilitating cellular signaling pathways (7). Dysfunction of NKA can have significant implications for insulin secretion and action, thereby contributing to T2D (5, 8). Recent studies have also linked NKA to diabetes complications like nephropathy and neuropathy (9, 10), with changes in its activity potentially exacerbating these conditions through oxidative stress and inflammation (11, 12). Additionally, NKA’s role in islet transplantation, where it may influence cell viability and immune responses, offers potential benefits for improving transplant outcomes. To develop new therapeutic approaches for T2D, it is essential to conduct in-depth research into the specific mechanisms governing NKA function and its regulation.

## 2 Biological function of NKA and its role in cellular function and metabolism

Structurally, NKA consists of two main subunits: the α subunit, which is responsible for the catalytic activity of the pump, and the β subunit, which aids in the proper maturation and localization of the enzyme within the membrane (13). NKA’s main role is to pump out three sodium ions and import two potassium ions per ATP molecule hydrolyzed, maintaining the cell’s membrane potential, which is vital for functions like muscle contraction and nerve signaling (13). This activity accounts for 30-40% of animal cells’ energy use, highlighting its importance in energy metabolism and electrolyte transport (14).

The regulation of NKA activity is complex and involves various mechanisms, including modulation of its expression levels, localization within the plasma membrane, enzymatic activity, and interactions with other proteins (14). NKA’s plasma membrane location is crucial for its antiport activity, with its movement between the membrane and intracellular spaces strictly regulated, although the precise mechanisms remain poorly understood (14).

NKA also functions as a signaling molecule, influencing various cellular pathways beyond ion transport. It regulates intracellular sodium and potassium concentrations, which significantly impact the flow of calcium ions and intracellular pH, thereby influencing signaling pathways critical for cell survival and function (15, 16). Additionally, NKA is linked to several metabolic pathways, including glucose transport and energy metabolism, which are essential for maintaining cellular homeostasis (14). Its interaction with signaling molecules allows it to influence pathways that regulate inflammation, cell growth, and apoptosis (17).

Research has shown that alterations in NKA activity can lead to various pathological conditions. For instance, impaired NKA function has been associated with diseases such as heart failure, where reduced NKA activity contributes to cardiac dysfunction and hypertrophy (18). Similarly, in the context of hyperuricemia, NKA signaling impairment has been linked to renal tubular injury, highlighting its role in kidney health (19, 20), Moreover, NKA’s involvement in regulating intracellular calcium levels is crucial for neuronal excitability and synaptic transmission, indicating its importance in the nervous system (6).

Overall, NKA is a multifunctional enzyme that is integral to maintaining cellular ion balance and facilitating various signaling processes. Its role extends beyond simple ion transport, influencing metabolic pathways and cellular responses to stressors. Understanding the complex regulatory mechanisms of NKA and its involvement in disease processes is essential for developing targeted therapies that can modulate its activity and improve cellular function in pathological conditions (17).

## 3 NKA ion transport mechanism in diabetic conditions

### 3.1 The association between NKA and diabetes

The relationship between NKA and diabetes is complex and multifaceted, involving alterations in the expression and activity of NKA that are closely linked to pancreatic function (21). In diabetes, research indicates a significant reduction in NKA activity (22, 23). The reduced activity of NKA can lead to an increase in intracellular sodium levels, which may disrupt cellular homeostasis and contribute to the dysfunction of pancreatic α-cells and β-cells.

Moreover, studies have highlighted the role of oxidative stress in T2D (24), which can further compromise NKA activity. Oxidative stress markers correlate with reduced NKA activity in various tissues (21). This suggests that the metabolic disturbances associated with T2D may be, in part, due to the inability of NKA to maintain ionic balance under oxidative conditions.

Furthermore, the relationship between NKA activity and metabolic syndrome (MS) is noteworthy, as individuals with T2D and MS exhibit significantly lower NKA activity compared to healthy controls (25). Decreased NKA activity correlates with higher oxidative stress, potentially promoting diabetes-related complications like cardiovascular disease (26). NKA dysfunction directly links to features of metabolic syndrome through several mechanisms. In obesity, reduced NKA activity leads to increased intracellular sodium levels, which can promote insulin resistance and hypertension (27–29). High sodium levels can also activate the renin-angiotensin-aldosterone system (RAAS), contributing to elevated blood pressure (29). By modulating NKA activity, it may be possible to address multiple features of metabolic syndrome, improving overall metabolic health.

The implications of NKA dysfunction extend beyond the pancreas. For instance, in diabetic peripheral neuropathy (DPN), which affects a substantial number of T2D patients, GLP-1 receptor agonist therapies have demonstrated improvements in nerve conduction and axonal excitability, potentially through the restoration of NKA function (30). This indicates that therapeutic strategies aimed at enhancing NKA activity may offer a protective effect against the neurological complications of diabetes. Additionally, the modulation of NKA activity has been implicated in the regulation of renal function in diabetes, as alterations in NKA expression and activity can lead to renal tubular dysfunction and contribute to the progression of diabetic kidney disease (31). Overall, the evidence suggests that the dysregulation of NKA is a key factor in the pathogenesis of T2D and its complications, highlighting the importance of further research into targeted therapies that could restore NKA function and improve metabolic outcomes in diabetic patients.

### 3.2 Role of NKA in islet β-cell and α-cell function and metabolism

NKA is essential for maintaining cellular ion balance, which is crucial for the proper function of pancreatic islet cells. In β-cells, NKA helps maintain membrane potential by regulating sodium and potassium ion levels, which is vital for calcium influx and insulin release (32). Impaired NKA function can disrupt this balance, leading to reduced insulin secretion and contributing to hyperglycemia. Similarly, in α-cells, NKA’s role in establishing ion gradients is critical for glucagon secretion. Reduced NKA activity can impair glucagon secretion, leading to dysregulated blood sugar levels. This dysfunction can make α-cells less sensitive to glucose changes, resulting in excessive glucagon secretion even at low blood sugar levels, which further disrupts glucose homeostasis.

NKA’s ion transport activity also plays a significant role in cellular metabolism and energy balance (13). It accounts for 20-25% of whole-body ATP consumption, highlighting its importance in energy metabolism (33). In disease conditions, NKA dysfunction can interfere with mitochondrial function and disrupt energy production (34). Additionally, NKA interacts with metabolic signaling pathways such as the AMPK pathway, which is crucial for cellular energy sensing and regulation (19, 33). Impaired NKA function can affect AMPK activity, disrupting cellular energy balance and contributing to metabolic disorders.

## 4 Biochemical pathways involved

### 4.1 Insulin secretion pathways

NKA regulates multiple aspects of insulin secretion by affecting membrane potential and calcium influx (32). For example, normal NKA activity promotes the opening of voltage-gated calcium channels, increasing intracellular calcium levels and activating the fusion and release of insulin secretion granules (32). Additionally, NKA interacts with the cAMP-PKA signaling pathway (35). When cAMP levels rise, PKA is activated, enhancing NKA activity and promoting insulin secretion (36).

### 4.2 Apoptosis pathways

NKA dysfunction triggers cell apoptosis via multiple mechanisms (37). One key mechanism involves the accumulation of intracellular sodium, which disrupts mitochondrial function and elevates reactive oxygen species (ROS) levels (37). The increase in ROS not only damages cellular macromolecules but also activates apoptotic pathways, such as the mitochondrial and endoplasmic reticulum stress pathways (37). Additionally, NKA dysfunction alters the balance of intracellular calcium, leading to the activation of proteases like calpain (38). This activation results in structural and functional damage to the cell, ultimately inducing apoptosis. These pathways underscore the critical role of NKA in maintaining cell viability and highlight the consequences of its dysfunction in the context of metabolic diseases like T2D.

### 4.3 Immune regulation pathways

NKA is involved in multiple signaling pathways in immune regulation. For example, NKA regulates the activity of the nuclear factor κB (NF-κB) signaling pathway by affecting intracellular sodium and calcium concentrations (39). NF-κB plays a key role in the activation, proliferation, and cytokine secretion of immune cells (40). Normal NKA activity inhibits the overactivation of the NF-κB signaling pathway, reducing the production of pro-inflammatory cytokines and alleviating inflammatory responses (39).

### 4.4 Oxidative stress

Oxidative stress is a central mechanistic factor in the dysfunction of NKA and the pathogenesis of T2D (24, 41). Elevated levels of oxidative markers such as malondialdehyde (MDA), protein carbonyls, and 8-hydroxy-2’-deoxyguanosine (8-OHdG) are directly linked to T2D (42). MDA, a product of lipid peroxidation, indicates damage to cell membranes and organelles, disrupting cellular homeostasis (43). Protein carbonyls reflect protein oxidation, which can alter protein structure and function, impairing NKA’s catalytic activity (44). 8-OHdG, a marker of DNA damage, suggests genomic instability and can affect the expression and function of NKA. These oxidative markers contribute to metabolic dysfunction by impairing insulin signaling, reducing β-cell function, and promoting insulin resistance (45).

## 5 The potential of NKA as a therapeutic target

Given the fundamental role of NKA in regulating insulin secretion and action, NKA is considered a promising therapeutic target for improving pancreatic function and insulin sensitivity in T2D patients. Research indicates that modulating NKA activity can lead to enhanced glucose homeostasis and improved metabolic outcomes (22, 46). For instance, specific antibodies targeting the NKA may stimulate its activity, leading to cardioprotective effects and improved metabolic profiles in models of chronic kidney disease and heart failure (47). Furthermore, the use of NKA activators has been associated with increased osteogenic differentiation in mesenchymal stem cells, suggesting a broader therapeutic potential for NKA modulation beyond metabolic diseases (48).

Recent investigations have also highlighted the significance of NKA in various cellular processes, including its role as a signaling molecule (6). NKA interacts with several proteins, influencing pathways that regulate cellular metabolism and proliferation (49). For example, the G protein-coupled receptor GPR35 has been shown to enhance NKA’s ion transport and signaling activity, which may have implications for T2D treatment (50). Additionally, the identification of endogenous negative regulators of NKA, such as inositol pyrophosphate 5-InsP7, suggests that manipulating these regulatory pathways could provide new strategies for enhancing NKA function in T2D (15).

Overall, the modulation of NKA presents a multifaceted approach to treating T2D and potentially other related conditions. The ongoing research into NKA’s role in cellular signaling, its interactions with various proteins, and its implications in metabolic and oncological contexts highlights its potential as a versatile therapeutic target. Continued exploration of NKA’s mechanisms and regulatory pathways will be essential in developing effective strategies to harness its therapeutic potential in clinical settings.

## 6 NKA activators, gene therapy, and stem cell therapy: novel approaches for treating T2D

Recent studies have highlighted the importance of NKA in the context of T2D and its potential as a therapeutic target. NKA dysfunction is linked to insulin resistance, β-cell failure, and increased oxidative stress, all of which contribute to the pathophysiology of T2D. Although there are no reports of direct applications of NKA activators in the treatment of T2D, existing findings suggest that modulating NKA activity may offer new insights for improving insulin secretion and restoring glucose homeostasis. Future research may further explore the potential applications of NKA activators in the treatment of T2D. Novel approaches including NKA activators, gene therapy, and stem cell therapy, can improve insulin secretion, and restore glucose homeostasis. These approaches offer novel therapeutic strategies that complement existing antidiabetic drugs and immunomodulatory therapies, providing a more comprehensive and updated perspective on the treatment of T2D.

### 6.1 NKA activators

NKA activators enhance NKA activity, which is crucial for cellular ion homeostasis and insulin secretion. The mechanism involves increasing NKA activity, which helps maintain intracellular sodium and potassium balance and promotes insulin release from pancreatic beta cells. This is beneficial for T2D patients with impaired insulin secretion. Specific NKA activators may improve glycemic control and insulin sensitivity, reducing blood glucose levels. However, potential side effects and off-target effects require careful monitoring and dosage control. NKA activators offer a novel therapeutic strategy for T2D.

### 6.2 Gene therapy

Gene therapy, especially using CRISPR/Cas9, targets genetic factors in T2D (51). It can edit genes associated with NKA function to restore normal activity, enhancing insulin secretion and glucose homeostasis. This is relevant in T2D, where ion transport dysregulation is a significant issue. Gene editing can correct mutations impairing NKA function, improving glucose responses and insulin sensitivity. Gene therapy can also deliver genes encoding beneficial proteins to enhance beta-cell function (52). Despite challenges like delivery methods and off-target effects, ongoing research explores its feasibility and efficacy, offering personalized and durable solutions for T2D.

### 6.3 Stem cell therapy

Stem cell therapy provides insulin-producing cells to restore pancreatic function in T2D (53). The differentiation of stem cells into pancreatic beta cells addresses the loss of insulin secretion (54). Advances in stem cell research have enabled the generation of functional pancreatic cells from PSCs, which can secrete insulin in response to glucose, potentially reversing hyperglycemia (55, 56). Adult pancreatic stem cells also show promise in regenerating damaged tissue and restoring insulin production (57). However, challenges include generating sufficient functional beta cells, managing tumorigenesis risk, and post-transplantation immunosuppression. Ongoing research aims to optimize differentiation protocols and explore combination therapies to improve T2D outcomes. Stem cell therapy is a promising regenerative approach to address the root causes of T2D.

## 7 Conclusion and future directions

**Figure 1:**
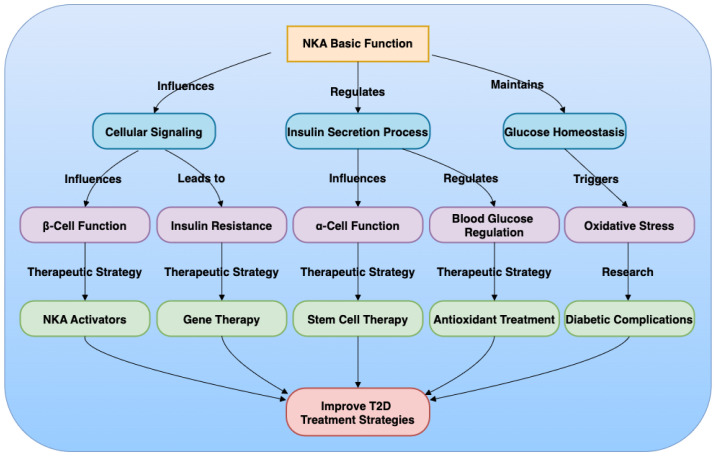
Mechanism of Na+/K+-ATPase (NKA) in Type 2 Diabetes (T2D).

This review has highlighted the multifaceted role of NKA in T2D, emphasizing its importance in cellular ion homeostasis, insulin secretion, and immune responses (**Figure 1**). The key points addressed include the biological functions of NKA, its involvement in T2D pathophysiology, and the potential of NKA as a therapeutic target. Areas warranting further investigation involve the development of more specific NKA modulators with fewer side effects and the application of experimental models to explore the clinical feasibility of targeting NKA. Future research should focus on refining NKA activators, gene therapy, and stem cell therapy to enhance their safety and efficacy. The development of novel experimental models, such as humanized mouse models and iPSC-derived islets, will be crucial for preclinical testing and translational research. In conclusion, modulating NKA activity offers promising therapeutic strategies for T2D, with the potential to improve glycemic control and enhance β-cell function. These strategies, if developed and applied effectively, could significantly impact the management of T2D and related complications, and may also contribute to improving outcomes in islet transplantation.
